# Loss of collagen content is localized near cartilage lesions on the day of injurious loading and intensified on day 12

**DOI:** 10.1002/jor.25975

**Published:** 2024-09-23

**Authors:** Moustafa Hamada, Atte S. A. Eskelinen, Cristina Florea, Santtu Mikkonen, Petteri Nieminen, Alan J. Grodzinsky, Petri Tanska, Rami K. Korhonen

**Affiliations:** ^1^ Department of Technical Physics University of Eastern Finland Kuopio Finland; ^2^ Institute of Biomedicine University of Eastern Finland Kuopio Finland; ^3^ Departments of Biological Engineering Electrical Engineering and Computer Science, and Mechanical Engineering, Massachusetts Institute of Technology Massachusetts Avenue Cambridge Massachusetts USA

**Keywords:** articular cartilage, collagen fibrils, inflammation, joint injury, post‐traumatic osteoarthritis

## Abstract

Joint injury can lead to articular cartilage damage, excessive inflammation, and post‐traumatic osteoarthritis (PTOA). Collagen is an essential component for cartilage function, yet current literature has limited understanding of how biochemical and biomechanical factors contribute to collagen loss in injured cartilage. Our aim was to investigate spatially dependent changes in collagen content and collagen integrity of injured cartilage, with an explant model of early‐stage PTOA. We subjected calf knee cartilage explants to combinations of injurious loading (INJ), interleukin‐1α‐challenge (IL) and physiological cyclic loading (CL). Using Fourier transform infrared microspectroscopy, collagen content (Amide I band) and collagen integrity (Amide II/1338 cm^−1^ ratio) were estimated on days 0 and 12 post‐injury. We found that INJ led to lower collagen content near lesions compared to intact regions on day 0 (*p* < 0.001). On day 12, near‐lesion collagen content was lower compared to day 0 (*p* < 0.05). Additionally, on day 12, INJ, IL, and INJ + IL groups exhibited lower collagen content along most of tissue depth compared to free‐swelling control group (*p* < 0.05). CL groups showed higher collagen content along most of tissue depth compared to corresponding groups without CL (*p* < 0.05). Immunohistochemical analysis revealed higher MMP‐1 and MMP‐3 staining intensities localized within cell lacunae in INJ group compared to CTRL group on day 0. Our results suggest that INJ causes rapid loss of collagen content near lesions, which is intensified on day 12. Additionally, CL could mitigate the loss of collagen content at intact regions after 12 days.

## Graphical abstract

We utilized Fourier transform infrared microspectroscopy to investigate spatial changes in collagen content and integrity in in vitro cultured cartilage explants after injury. Our results suggest that injurious loading could cause rapid collagen content loss in cartilage localized near lesion. Additionally, the application of physiological cyclic loading after injury could mitigate later collagen loss along cartilage tissue depth caused by injurious loading and/or inflammation after 12 days.

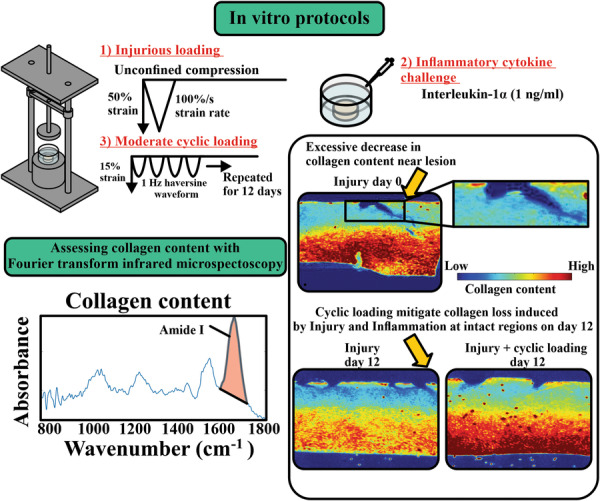

## 1 INTRODUCTION

The collagen fibril network is a central part of the articular cartilage structure.^1^, ^2^, ^3^ This fibrillar matrix not only provides the necessary tensile and shear stiffness for the tissue, but also plays a crucial role in resisting the swelling pressure induced by the negatively charged proteoglycans providing compressive stiffness to the tissue.^3^, ^4^ Traumatic loading and possible aggressive inflammatory responses upon joint injury can disturb cartilage homeostasis and lead to damage to vital tissue components, such as the collagen fibril network.^5^, ^6^, ^7^ Articular cartilage has extremely limited repair capability, thus cartilage damage following the injury is often permanent.^8^ The cartilage damage not only jeopardizes its function but can disrupt the normal functions of the other joint tissues, ultimately causing post‐traumatic osteoarthritis (PTOA), a disease that mainly affects young, active individuals.^8^, ^9^, ^10^

In vitro models of PTOA have been used to capture the nature of tissue degeneration after cartilage injury.^6^, ^11^, ^12^, ^13^, ^14^ Previous studies have suggested that mechanical overloading can cause collagen denaturation, a decrease in collagen and proteoglycan biosynthesis, proteoglycan loss, and cell death.^6^, ^13^, ^14^, ^15^ On the other hand, studies^13^, ^15^, ^16^ have also shown that tissue degradation is linked with the infiltration of pro‐inflammatory cytokines, such as interleukin (IL)‐1, IL‐6, and tumor necrosis factor (TNF)‐α, into the cartilage tissue leading to damage of extracellular matrix via upregulation of matrix metalloproteinases (MMPs)^16^, ^17^ and aggrecanases.^17^, ^18^ However, most of the previous studies have only focused on bulk changes of the collagen fibrils or proteoglycans, while little is known about the localization of collagen fibril damage progression within the tissue after mechanical overloading and inflammation.^5^, ^6^, ^7^, ^19^, ^20^, ^21^

Fourier transform infrared (FTIR) microspectroscopy has become a popular tool in studying spatially dependent molecular changes in soft tissues such as cartilage.^22^, ^23^, ^24^, ^25^, ^26^, ^27^ Previous studies have found a direct association between Amide I absorbance band (1580–1720 cm^−1^, C=O stretching vibration) and cartilage collagen content.^22^, ^23^, ^24^, ^27^ Additionally, previous studies have reported that the ratio between the integrated areas under the Amide II absorbance band (1485–1585 cm^−1^, C–N stretching vibration^27^) and the absorbance band centered at 1338 cm^−1^ (1300–1356 cm^−1^, CH_2_ side‐chain vibrations in collagen fibrils) increases when collagen fibrils are denatured with collagenases.^22^ This ratio parameter is often considered as a measure of collagen integrity.^22^, ^23^ By analyzing collagen content and collagen integrity, one can determine whether the nature of the early collagen network damage in injured and inflamed cartilage is compositional (i.e., loss of collagen content), molecular (i.e., loss of structural quality of the collagens fibrils), or both.

In our previous in vitro study, we investigated spatial changes of aggrecan content in injuriously loaded cartilage explants that were subjected to combinations of IL‐1α inflammatory cytokine challenge and moderate cyclic loading for 12 days.^13^ These protocols were used to mimic the biomechanical and biochemical environment of cartilage after traumatic injury. We observed lower aggrecan content in injuriously loaded and IL‐1‐treated cartilage groups compared to the free‐swelling control group already after 3 days, localized especially in the vicinity of lesions. Additionally, we observed cell death localized near lesions in the sections of injuriously loaded explants. By day 12, cell death was observed to expand to deeper cartilage regions.^13^ In the current study, we aim to assess possible early spatial changes in collagen content and collagen integrity in the same in vitro model on days 0 and 12. We hypothesized that injurious loading would result in localized loss of collagen content near the lesion compared to the non‐injured explant region. Additionally, the combination of injurious loading, inflammation and cyclic loading was hypothesized to potentiate the decrease of collagen content and integrity.^13^, ^28^ Our hypothesis was motivated by previous studies showing evidence that collagen fibrils undergo alterations near the overloaded area caused by direct mechanical rupture of collagen fibrils and/or enzymatic degradation triggered by cell damage,^5^, ^6^, ^14^, ^29^, ^30^ with presumably more MMP‐driven cleavage of collagen fibrils in injured/inflamed cartilage than in untreated cartilage. With this experimental explant model, FTIR microspectroscopy, and immunohistochemical (IHC) staining of MMPs, we aim to extend understanding of the cartilage compositional changes underlying the progression of PTOA at the early stage of the disease.

## 2 METHODS

### 2.1 Sample preparation and study design

**Figure 1:**
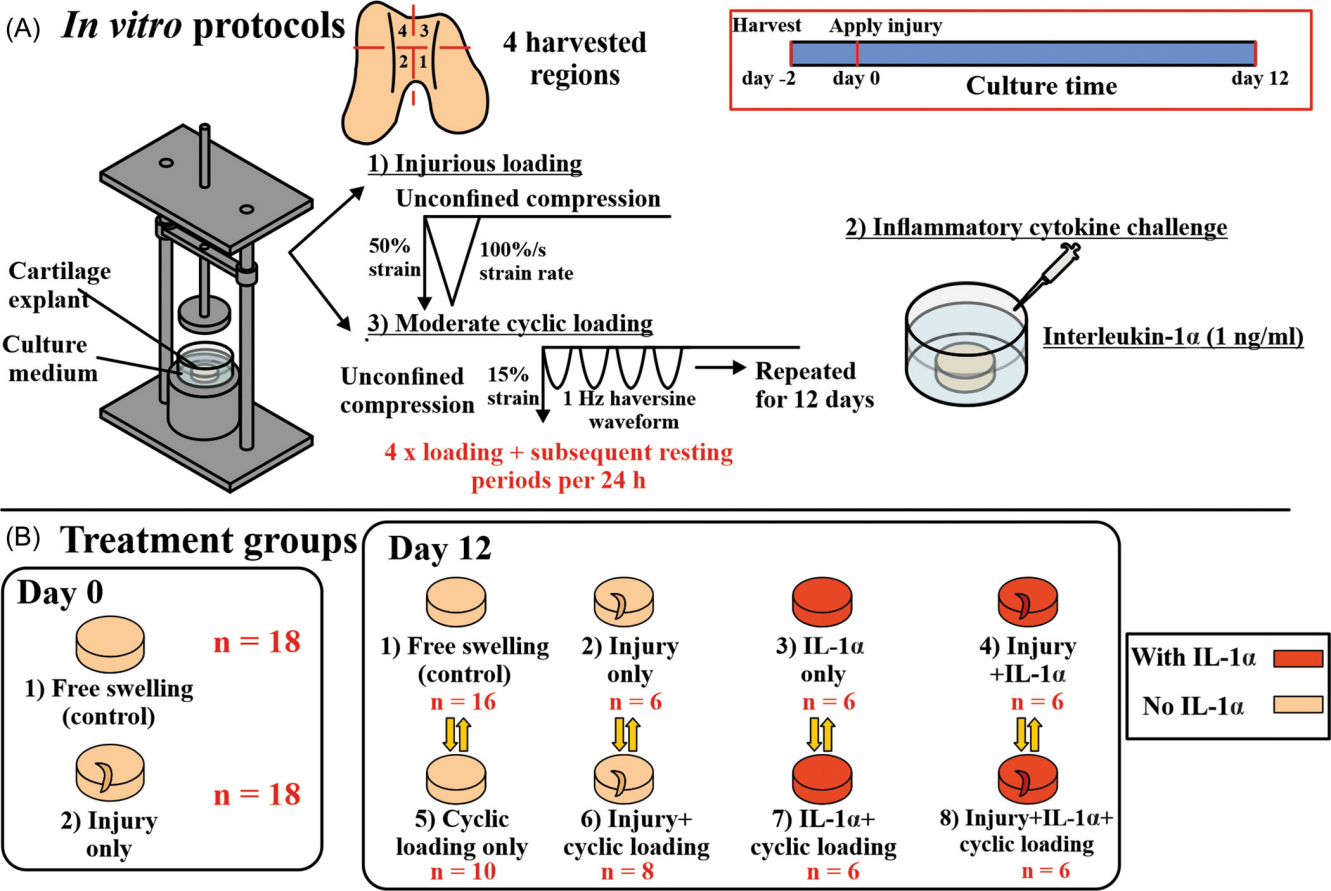
Study design and treatment groups. (A) Cartilage explants (*n* = 105) were harvested in two experiments from the patellofemoral grooves of young bovine knee joints (*N* = 11) and equilibrated in the culture medium for 2 days. Next, each explant was subjected to 1) injurious loading, 2) pro‐inflammatory IL‐1α‐challenge, 3) moderate cyclic loading, or a combination of these. The explant culture was terminated either on day 0 or day 12. (B) Cartilage explants were divided into groups of the different treatments.

This study combines cultured cartilage explants from two previous sets of experiments with similar design which focused on localized aggrecan loss near cartilage injury.^13^, ^31^ Here, we focus on collagen content changes that were not investigated earlier. Cartilage explants (*n* = 105) were obtained from four regions within the patellofemoral groove of a single knee joint in each of 11 newborn bovines (Figure 1A). The patellofemoral grooves were used due to their flat surfaces, facilitating the extraction of numerous intact cylindrical explants. Immature cartilage explants were used since immature tissues exhibit minimal variation in the biochemical and biomechanical properties compared to mature tissues, ensuring repeatability of cartilage responses in this experimental explant model across different treatment conditions. All explants were prepared using a 3‐mm dermal punch, and then trimmed to 1‐mm thickness with a razor blade. Explant culture conditions were done as described in more detail previously.^13^, ^31^ The explants were subjected to various experimental conditions (see Section 2.2 for more details): 1) injurious loading (INJ), 2) IL‐1α challenge (IL), 3) moderate cyclic loading (CL), or their combinations (Figure 1B). Additionally, a set of cartilage explants was subjected to free‐swelling condition as control group (CTRL). Explants for each treatment group were region‐matched with their corresponding control explants.

In our experiment (Experiment #1)^13^, we applied the cyclic loading protocol with IL‐1α and their combination with injurious loading (IL + CL and INJ + IL + CL groups).^13^ However, we also aimed to assess the effect of physiological cyclic loading on injurious loading alone. To achieve this, we utilized cartilage explants from our previous experiment (Experiment #2)^31^ and introduced two new groups (CL and INJ + CL groups). The culture was terminated either on the same day of injury (day 0) for CTRL and INJ explants or on day 12 for CTRL and all treatment combinations (INJ, IL, CL, INJ + IL, INJ + CL, IL + CL, and INJ + IL + CL).

### 2.2 In vitro protocols

#### 2.2.1 Injurious loading

After the 2‐day equilibration period, the explants assigned to injuriously loaded groups were subjected to a single load–unload cycle of unconfined compression (50% strain amplitude, 100%/s strain rate) using a custom‐designed incubator‐housed loading apparatus (Figure 1A).^32^ This and similar loading protocols (similar strain amplitude and strain rate^33^) have been shown to result in the formation of visible superficial chondral lesions on average in ~80% of explants subjected to injurious loading.^33^

#### 2.2.2 Inflammatory cytokine challenge

To simulate joint inflammation and the subsequent biochemical responses after the joint injury, a concentration of 1 ng/ml of exogenous pro‐inflammatory cytokine IL‐1α was added to the culture medium of explants that were assigned to IL groups, allowing the cytokines to diffuse into the tissue. The selection of the IL‐1α concentration was chosen based on prior dose–response tests^13^, ^15^, ^34^ and it resulted in PTOA‐like matrix loss in such a manner that inflammation‐driven matrix loss would not dominate over injury‐driven matrix loss.^13^, ^15^, ^17^ We opted for IL‐1α over IL‐1β, as it has been shown that IL‐1α is more potent in causing matrix loss in bovine cartilage.^35^

#### 2.2.3 Moderate cyclic loading

Cartilage explants assigned to cyclically loaded groups were placed in a multi‐well polysulfone loading chamber.^13^, ^31^ First, contact between the cartilage surface and the compressive platen was ensured by a 10% pre‐strain in unconfined compression. Subsequently, loading with a 15% strain amplitude at 1 Hz frequency of haversine waveform with a 40% duty cycle was applied (Figure 1A). The protocol was designed to mimic physiological patterns of cyclic loading.^13^, ^20^, ^31^ In both Experiment #1 and Experiment #2, cyclic loading was implemented with a 1‐h continuous loading followed by four subsequent resting periods. The primary difference between the two experiments was in the resting periods. In Experiment #1, the resting periods were of 3 h, 4 h, 3 h, and 10 h (an overnight rest) for every 24‐h period (i.e., the explant was unloaded).^13^ However, in Experiment #2, each resting period lasted 5 h for every 24‐h period.^31^ In our previous studies, both protocols have been tested and suggested to be within healthy physiological range^13^, ^31^ (see the discussion for more details on the protocols).

### 2.3 Fourier transform infrared microspectroscopy measurements

**Figure 2:**
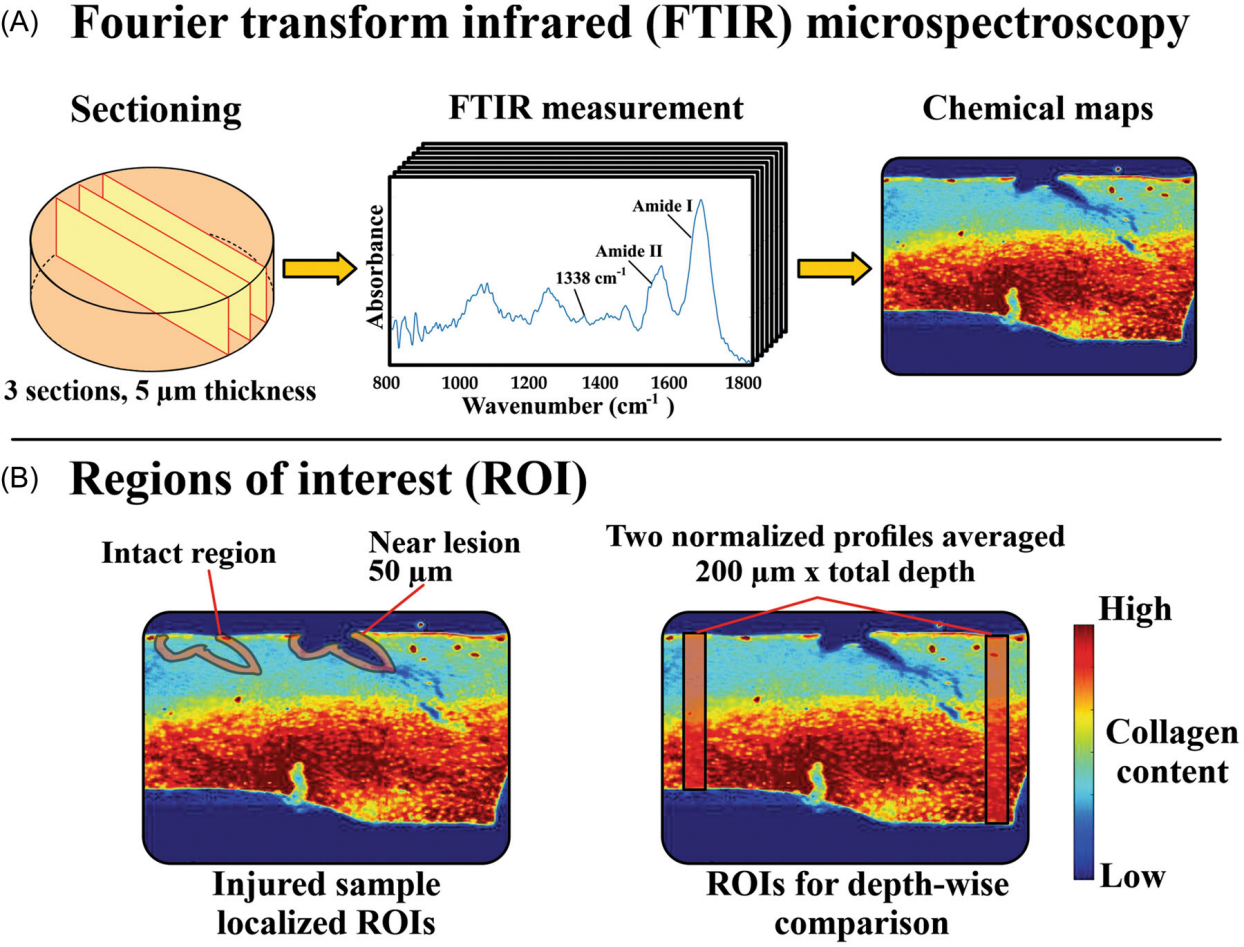
Fourier‐transform infrared microspectroscopic measurements and regions of interest (ROI). (A) Three 5 µm cartilage sections were sliced with microtome from each explant. Spectra were obtained point by point from each section (5.5 × 5.5 µm^2^ spatial resolution) within the post‐truncated wavenumber range of 800–1800 cm^−1^. After spectral processing (see Figure 3), chemical maps were obtained for further analysis. (B) For injured groups only, collagen content and collagen integrity were assessed locally by defining a ROI near the lesion with 50 µm distance from lesion edges, and then the same ROI geometry was translated into adjacent intact region. Second, for all treatment groups, collagen content was assessed depth‐wise by defining two ROIs (200 µm × section depth) at two separate intact regions of cartilage. The two ROIs are then averaged to obtain one averaged normalized profile for each explant (0% = top, 100% = bottom).

After the termination of experiments, all explants were fixed in formalin and decalcified for later assessment of the effect of each treatment on the collagen content and collagen integrity. Each explant was dehydrated with graded series of alcohols, transferred to intermediary xylene, and embedded in paraffin. Next, three 5 µm‐thick histological sections perpendicular to the articular surface were assigned for FTIR measurements. The three sections were cut from the middle of the explants using a microtome (Figure 2A). After the removal of paraffin, the sections were attached to Barium‐Fluoride (BaF_2_) windows that are near‐transparent to mid‐infrared light.^27^

The measurements were then conducted using an FTIR microspectroscopy system (Agilent Cary 670/620; Agilent Technologies, Santa Clara, CA, USA) equipped with a focal plane array detector (field of view: 140 × 140 µm^2^) and an optical microscope. Measurements were done in the transmission mode with a spatial pixel size of 5.5 × 5.5 µm^2^, spectral resolution of 4 cm^−1^, and wavenumber range of 750–3800 cm^−1^. To increase the signal‐to‐noise ratio, 8 repeated scans per pixel were taken. Before starting the measurements, a background scan was initially performed on a clean BaF_2_ window for calibration.

#### 2.3.1 Data pre‐processing and regions of interest

The acquired spectral data was processed using MATLAB (R2022b, MathWorks Inc., MA, USA). First, the original spectrum was truncated to a narrower wavenumber range (800–1800 cm^−1^). To assess spatial‐dependent changes in collagen content and integrity (see subsection 2.3.2) between and within groups, regions of interest (ROIs) were defined in two sets of assessments (near lesion vs. away from lesion and depth‐dependent). In the first assessment, collagen content and integrity near the lesions were compared to the intact region away from the lesion within the same explant (Figure 2B). Accordingly, this assessment included only the injuriously loaded groups. After assessing three sections per explant, 75% of the explants displayed visible chondral lesions. The explants without visible lesions were excluded from the analysis (11 out of 44). In each section, two regions of interest (ROIs) were established. The first ROI was defined near lesion within 50 µm from the edges of the lesion, while the second ROI with the same geometry was defined in an intact region away from the lesion. For each section per explant, average collagen content and integrity values were obtained from both ROIs, and then averaged to obtain estimates of the two parameters near‐lesion and away‐from‐lesion regions at each explant. Then, normalized collagen content and integrity values were calculated for each section by normalizing the value obtained from near‐lesion ROI to the corresponding value obtained from away‐from‐lesion ROI. This was done to obtain explant‐specific estimates and account for the biological variations (i.e., explants harvested from different animals and different locations) between the explants.

In the second assessment, two full‐thickness and 200 µm‐wide ROIs were first defined for each section totaling six ROIs per explant (Figure 2B). These ROIs were first averaged in the lateral ROI direction and interpolated to a 100‐point long vector in the axial direction to form six depth‐wise collagen content profiles. Finally, these six profiles were averaged to form a depth‐dependent normalized profile for each explant (0% = surface, 100% = bottom).

#### 2.3.2 Collagen content and collagen integrity assessments

**Figure 3:**
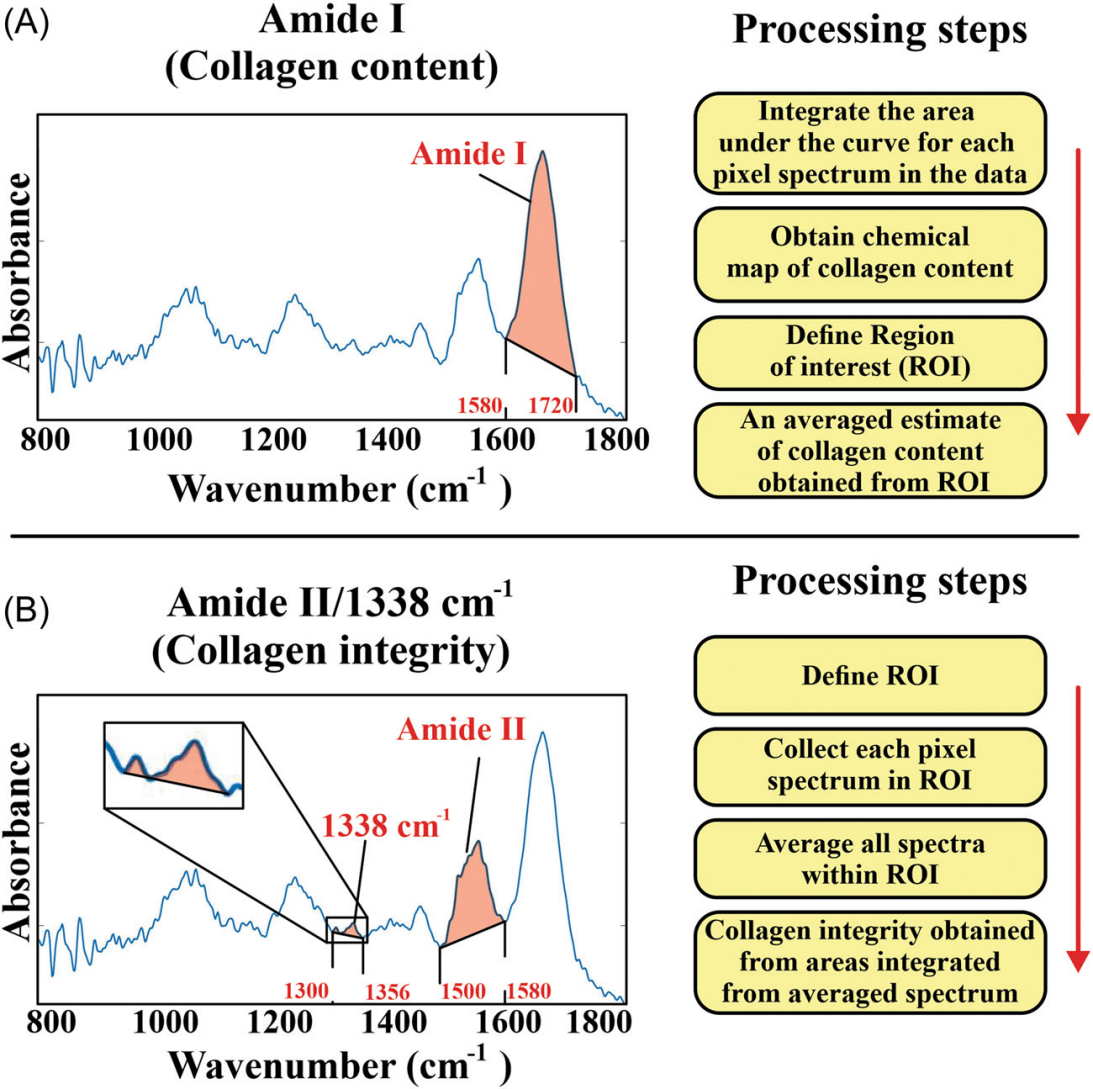
Processing of Fourier transform infrared spectra. To obtain collagen content and integrity values, the borders of Amide I, 1338 cm^−1^, and Amide II regions were defined, respectively. A linear baseline correction was conducted and the area under each region was calculated. (A) For collagen content, the assessment was done by processing the spectrum of each pixel. The obtained pixel‐wise collagen content values were then averaged in each region of interest (ROI). (B) For collagen integrity assessment, the spectra from pixels in each ROI were averaged and the averaged spectrum was baseline corrected. Next, the Amide II and 1338 cm^−1^ areas under the curve were calculated. The ratio Amide II/1338 cm^−1^ was used as an estimation of collagen integrity (higher value = decrease in collagen integrity, lower value = increase in collagen integrity).

To obtain an estimate for collagen content, we first defined the Amide I absorbance band (1580–1720 cm^−1^) in the spectrum in each pixel followed by linear baseline correction and calculation of the area under the Amide I curve (Figure 3A).^27^, ^36^, ^37^ We used these pixel values to create chemical maps for collagen content in each section (Figure 2A).

We estimated collagen integrity by the following procedure. Due to the low signal‐to‐noise ratio of the 1338 cm^−1^ absorbance band (Figure 3B), all pixels’ spectrums within ROI were averaged over each wavenumber to obtain an average spectrum for the entire ROI. Then, Amide II and 1338 cm^−1^ absorbance bands (1485–1585 cm^−1^ and 1300–1356 cm^−1^, respectively) were defined from the average spectrum followed by linear baseline correction and calculation of the area under the Amide II and 1338 cm^−1^ bands. Finally, to avoid errors due to concentration‐dependent changes in the spectra, the Amide II/1338 cm^−1^ ratio was calculated to form an estimate of collagen integrity in each ROI.^22^, ^23^, ^25^ An increase in this ratio would indicate a decrease in collagen integrity.^22^, ^25^

### 2.4 Immunohistochemical staining

To qualitatively assess the presence and localization of MMP‐1 and MMP‐3 in response to injurious loading and IL‐1α treatment, IHC analysis was performed. These proteases are associated with enzymatic cleavage of collagen fibrils after cartilage damage both ex vivo and in vivo.^38^, ^39^ The IHC was done using antibody detection kits (Biomatik, Ontario, Canada) specific for reactivity with bovine tissues. For each protease, two 4‐µm‐thick sections were used from each of the following treatment groups: day 0 (CTRL and INJ) and day 12 (CTRL, INJ, and IL). First, deparaffinizing of the sections was done using xylene and rehydration was done using ethanol. Next, for antigen retrieval, the sections were subjected to citrate buffer for 15 min (0.01 M, pH 6.0). To remove endogenous peroxidase activity, sections were treated with 1% H_2_O_2_ and washed with water. To block nonspecific staining, sections were treated with 1% bovine serum albumin (30 min, 37^ο^C). Subsequently, sections were incubated with primary antibodies (1:25 dilution for MMP‐1 and MMP‐3) overnight at 4^ο^C. On the following day, sections were incubated for 1 h with secondary antibody followed by diaminobenzidine for 5 min and counterstaining with hematoxylin for 1 min. For validation of staining protocol, positive and negative control sections were used (human gallbladder tissue for MMP‐1 and human kidney tissue for MMP‐3). Imaging of the stained sectionfs was done using light microscope (Zeiss Primostar 3, Jena, Germany) with magnifications of 4×, 10×, 40×, and 100×.

### 2.5 Statistical analysis

Statistical analyses were done with IBM SPSS Statistics 29.0 (SPSS Inc., IBM Company, Armonk, NY, USA). Considering that explants were harvested from different animals and regions on the articular surfaces, we utilized linear mixed effects (LME) model to statistically test the differences in collagen content and integrity between and within groups.

In the LME model, the animals (*N* = 11) were considered as the subject with random intercepts to account for the biological variations between the animals. Additionally, we used a diagonal covariance structure in the models to account for the variation in the number of explants at each treatment group. First, for the near‐lesion and away‐from‐lesion collagen content and integrity assessments within and between groups, treatment and section‐wise ROIs (i.e., near and away from lesion) were defined as fixed factors. Second, in the depth‐dependent profile assessments between treatment groups, treatment was the only fixed factor. When comparing the profiles of two treatment groups, at each normalized depth, if the LME‐predicted mean for collagen content value of one group fell outside the predicted 95% confidence intervals (CIs) of another group, the difference between the two groups was considered statistically significant (*p* < 0.05) at that point of normalized depth. Additionally, when comparing the two groups, the results were further validated with confidence intervals for difference in means. For all the comparisons between and within treatment groups, the significance level was set to *α* = 0.05, and the post‐hoc analysis was done using Fisher's Least Significant Difference.

## 3 RESULTS

### 3.1 Near‐lesion collagen content and integrity analysis

**Figure 4:**
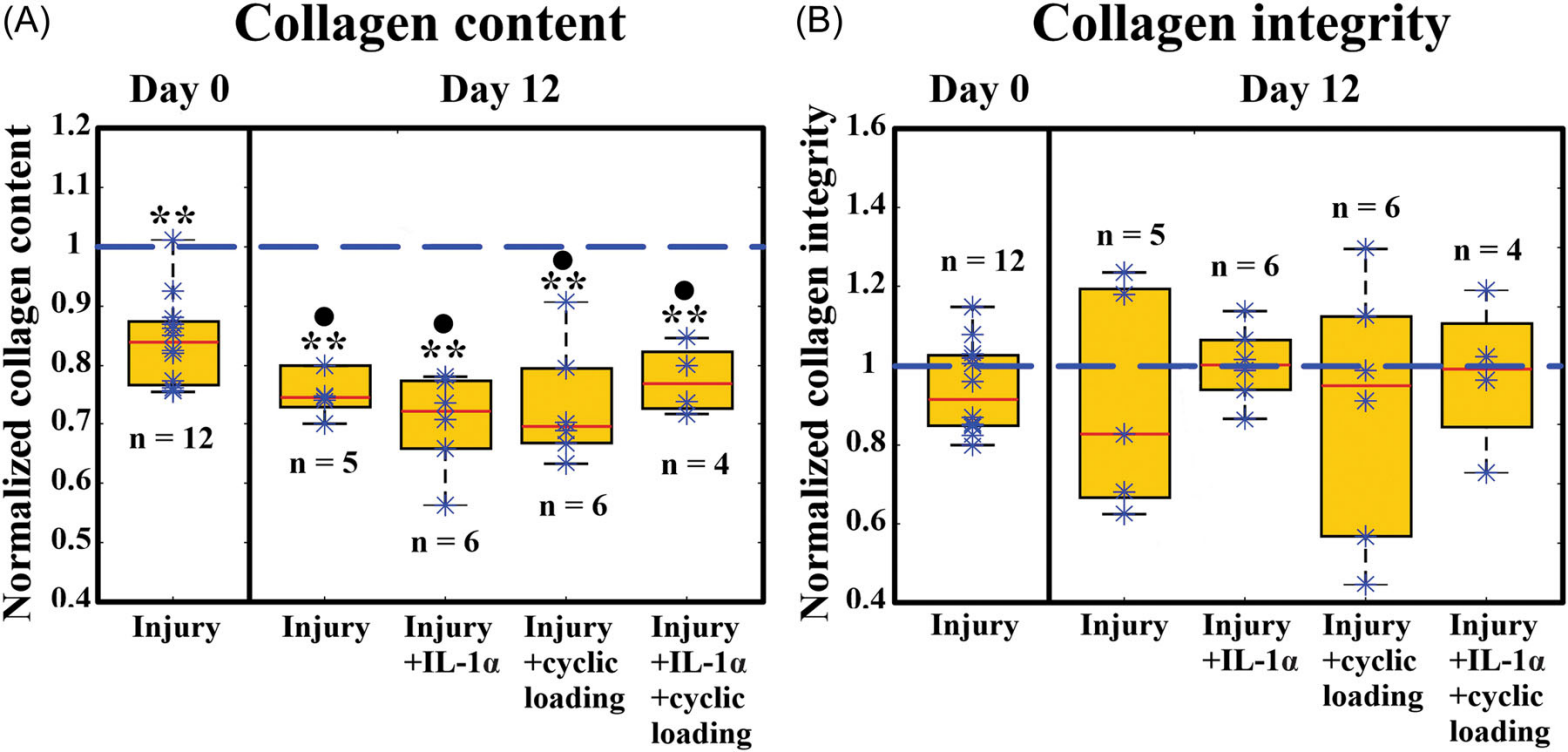
Collagen content and integrity near‐lesion normalized to away from lesion within injured groups. (A) Collagen content near‐lesion was significantly smaller in all injured groups on day 0 and day 12 (*p* < 0.001). Comparing between the two time points, all injured groups on day 12 had smaller normalized collagen content compared to day 0. (B) Normalized collagen integrity showed no alterations within the groups or between the two timepoints. *n* indicates the number of explants used per group. In these plots, a normalized value below one means that the near‐lesion collagen content or integrity was lower than the corresponding away‐from‐lesion value. ** = compared to away from lesion (that is, normalized value of one; *p* < 0.001), ● = compared to normalized collagen content on day 0. The box plots show median and quartile values. A linear mixed effects model with Fisher's Least Significant Difference was used for the comparisons between groups.

The analysis within INJ group showed that the collagen content was on average 14% lower near the lesion compared to away from lesion on day 0 (95% CI: 9–19%, *p* < 0.001, Figure 4A).

On day 12, the collagen content near the lesion, compared to away from lesion, was further decreased in all injury groups (INJ: 26% (95% CI: 21–31%), INJ + IL: 28% (95% CI: 20–36%), INJ + CL: 29% (95% CI: 22–36%), and INJ + IL + CL: 22% (95% CI: 9–35%), *p* < 0.001, Figure 4A). On the other hand, no significant differences in the normalized collagen integrity (near to/away from lesion) were found within groups or between the two time points (Figure 4B).

### 3.2 Depth‐wise collagen content analysis

**Figure 5:**
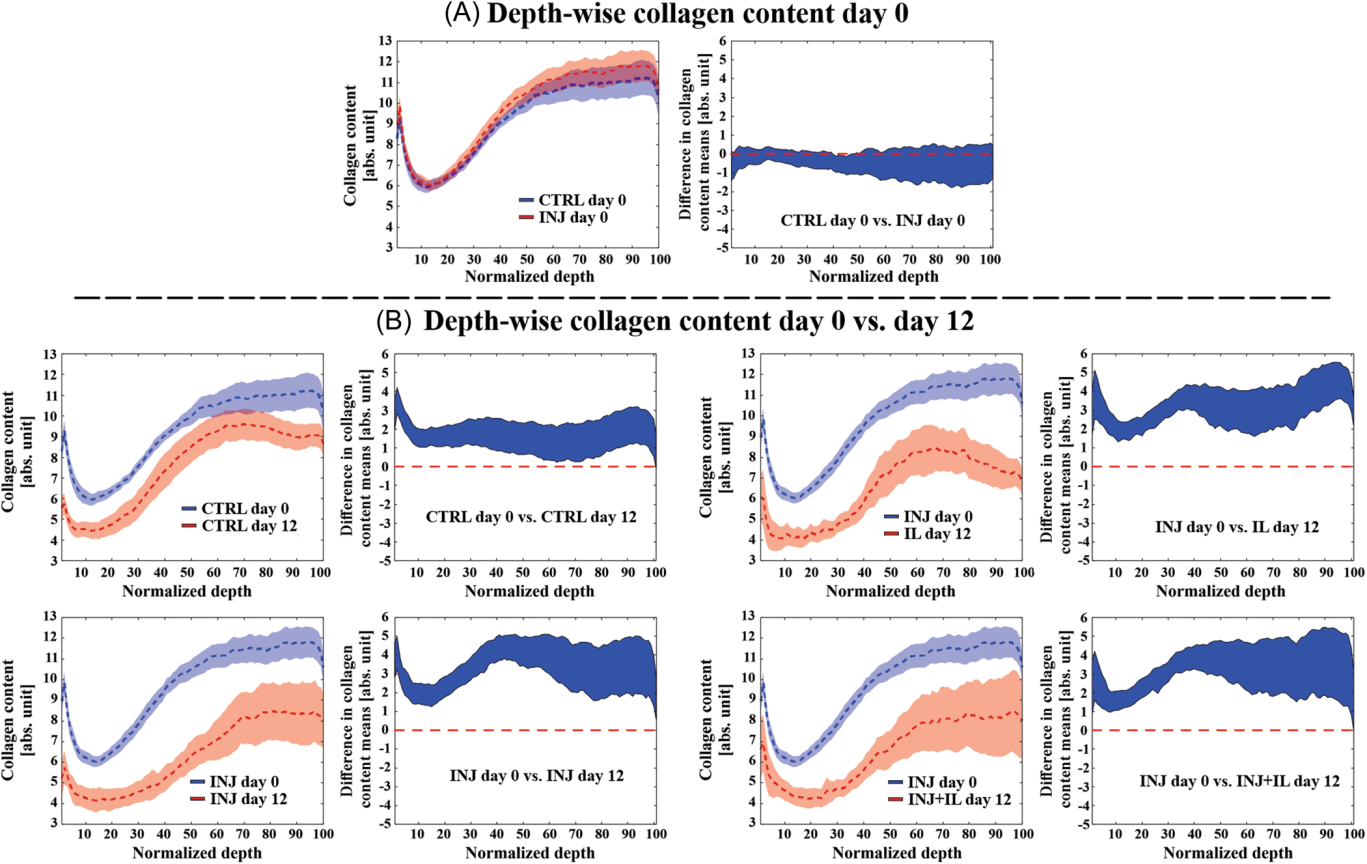
Depth‐wise collagen content profiles between day 0 and day 12 groups. (A) No differences between INJ and CTRL groups at most of tissue normalized depth on day 0. (B) CTRL and INJ groups on day 0 showed higher collagen content along whole normalized depth compared to CTRL, INJ, IL, and INJ + IL groups on day 12. For each pairwise comparisons, the left figure indicates the mean values (±confidence intervals, CI), and the right figure indicates a 95% CI for difference between means. Linear mixed effects model was utilized for the statistical comparisons. If the mean value of one group fell outside the 95% confidence intervals of another group, the difference between the two groups was considered statistically significant. *p* < 0.05, CTRL, free‐swelling control; INJ, injurious loading; IL, interleukin‐1α.

**Figure 6:**
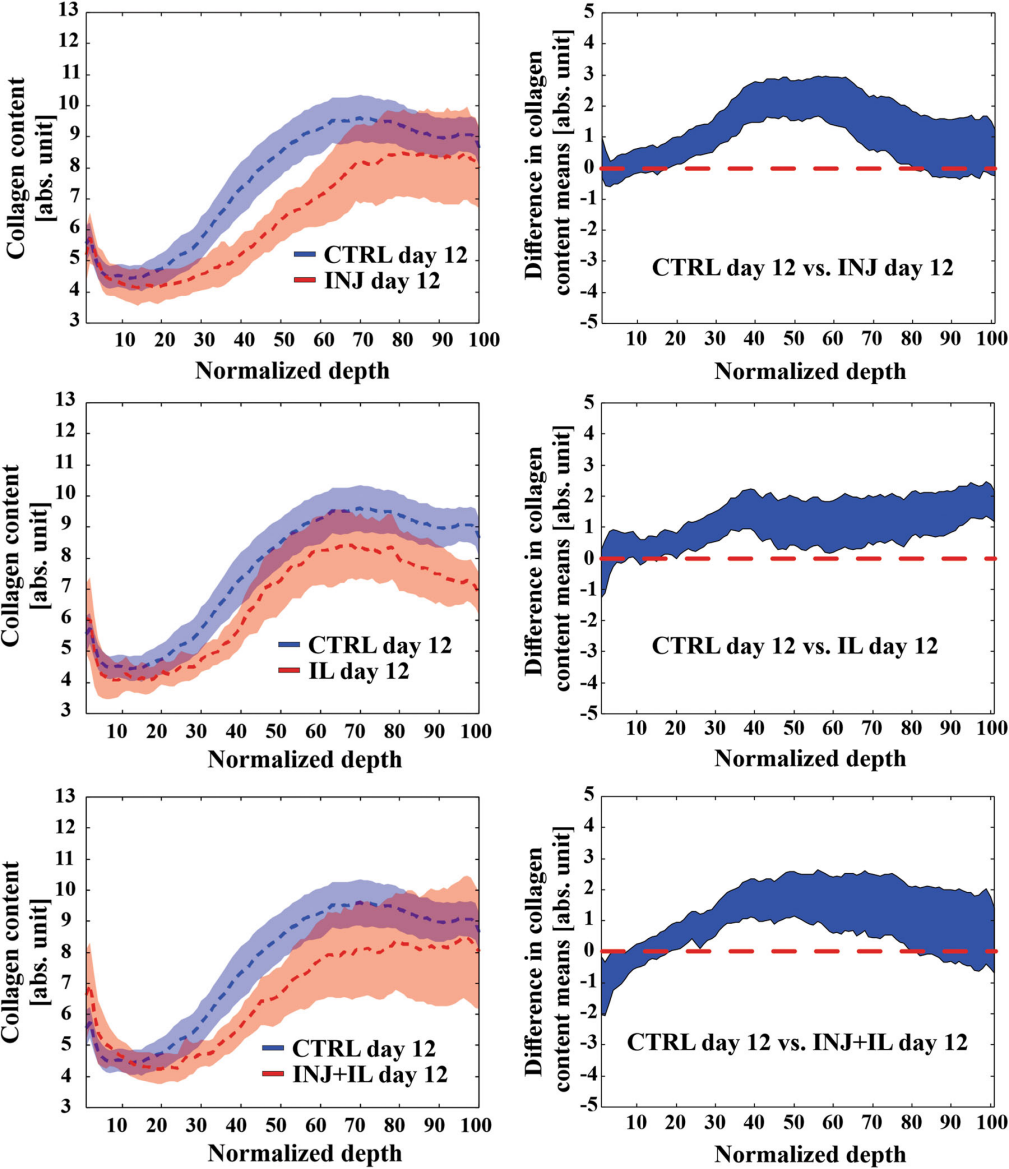
Depth‐wise collagen content between control, injured and inflamed groups on day 12. INJ, IL, and INJ + IL groups exhibited lower collagen content compared to CTRL group throughout most of tissue depth. For each pairwise comparisons, the left figure indicates the mean values (±cfonfidence intervals, CI), and the right figure indicated a 95% CI for difference between means. Linear mixed effects model was utilized for the statistical comparisons. If the mean value of one group fell outside the 95% confidence intervals of another group, the difference between the two groups was considered to be statistically significant. *p* < 0.05, CTRL, free‐swelling control; INJ, injurious loading; IL, interleukin‐1α.

CTRL, INJ, IL, and INJ+IL groups on day 12 exhibited lower collagen content when compared to CTRL and INJ groups on day 0 (0–100% of normalized depth, *p* < 0.05, Figure 5). On day 12, LME models showed that INJ, and IL groups had lower collagen content at most of normalized tissue depths compared to CTRL group (~19–74% and 18–100%, respectively, *p* < 0.05, Figure 6). Additionally, INJ + IL group exhibited lower collagen content compared to CTRL group at 20–76% of normalized depth (*p* < 0.05, Figure 6). However, no statistical differences were found between INJ, IL, and INJ + IL groups.

**Figure 7:**
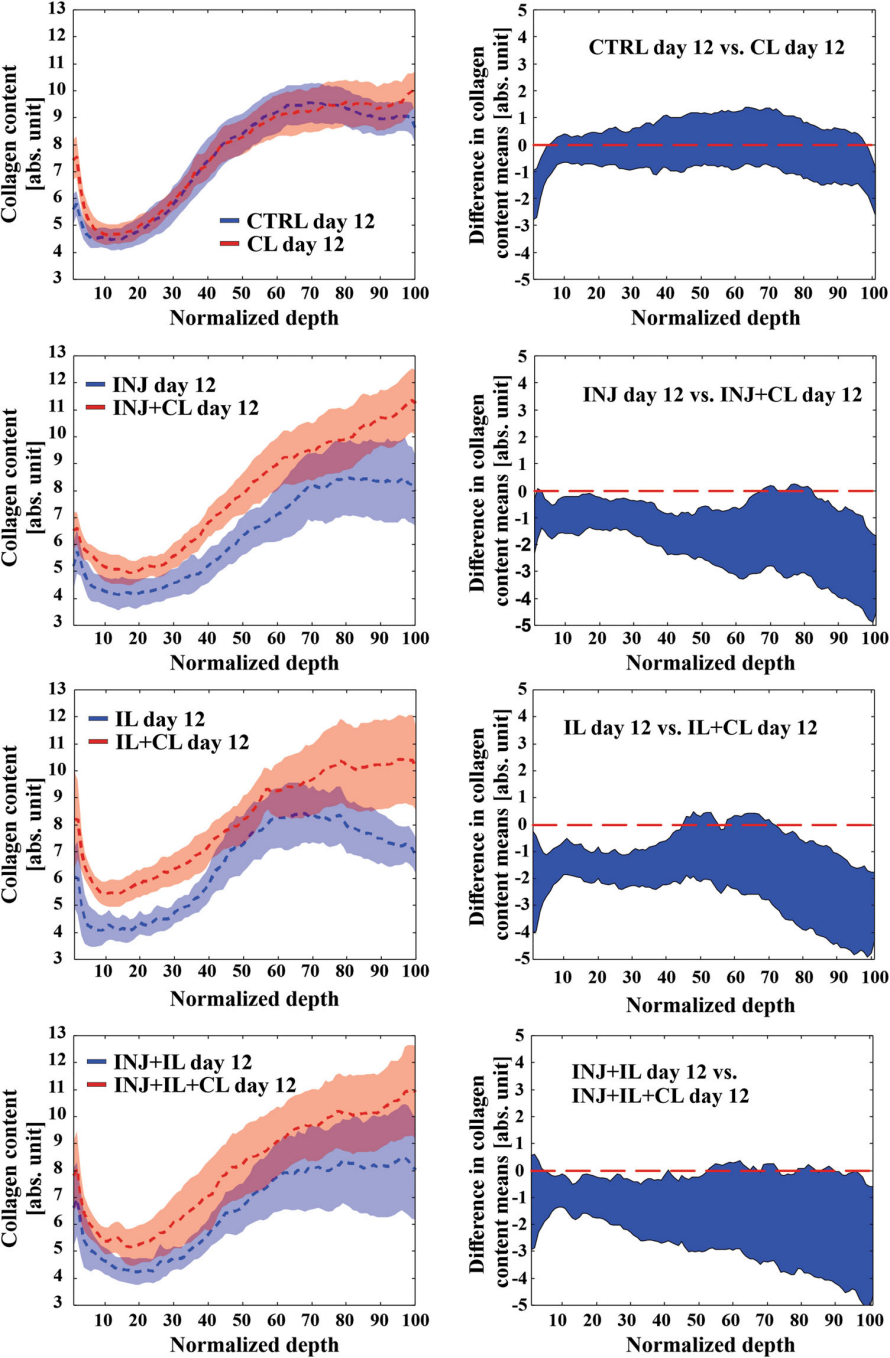
Depth‐wise collagen content between cyclic loading groups and groups without cyclic loading on day 12. No difference was found in collagen content along whole depth between CTRL and CL groups. However, INJ + CL, IL + CL, and INJ + IL + CL show higher collagen content along whole depth compared to INJ, IL, and INJ + IL, respectively. For each pairwise comparisons, the left figure indicates the mean values (± confidence intervals, CI), and the right figure indicated a 95% CI for difference between means. Linear mixed effects model was utilized for the statistical comparisons. If the mean value of one group fell outside the 95% confidence intervals of another group, the difference between the two groups was considered to be statistically significant. *p* < 0.05, CTRL, free‐swelling control; INJ, injurious loading; IL, interleukin‐1α; CL, cyclic loading.

When considering cyclic loading, INJ + CL and IL + CL groups exhibited higher collagen content almost throughout normalized tissue depth compared to INJ and IL groups, respectively (*p* < 0.05, Figure 7). Likewise, INJ + IL + CL group had higher collagen content at most of normalized tissue depths compared to INJ + IL group (*p* < 0.05, Figure 7). No differences were found on day 12 between CL and CTRL groups along the whole tissue depth. Lastly, when comparing collagen content between CL groups, we found no significant difference along the normalized depth between any of the groups.

### 3.3 MMPs immunohistochemical staining

**Figure 8:**
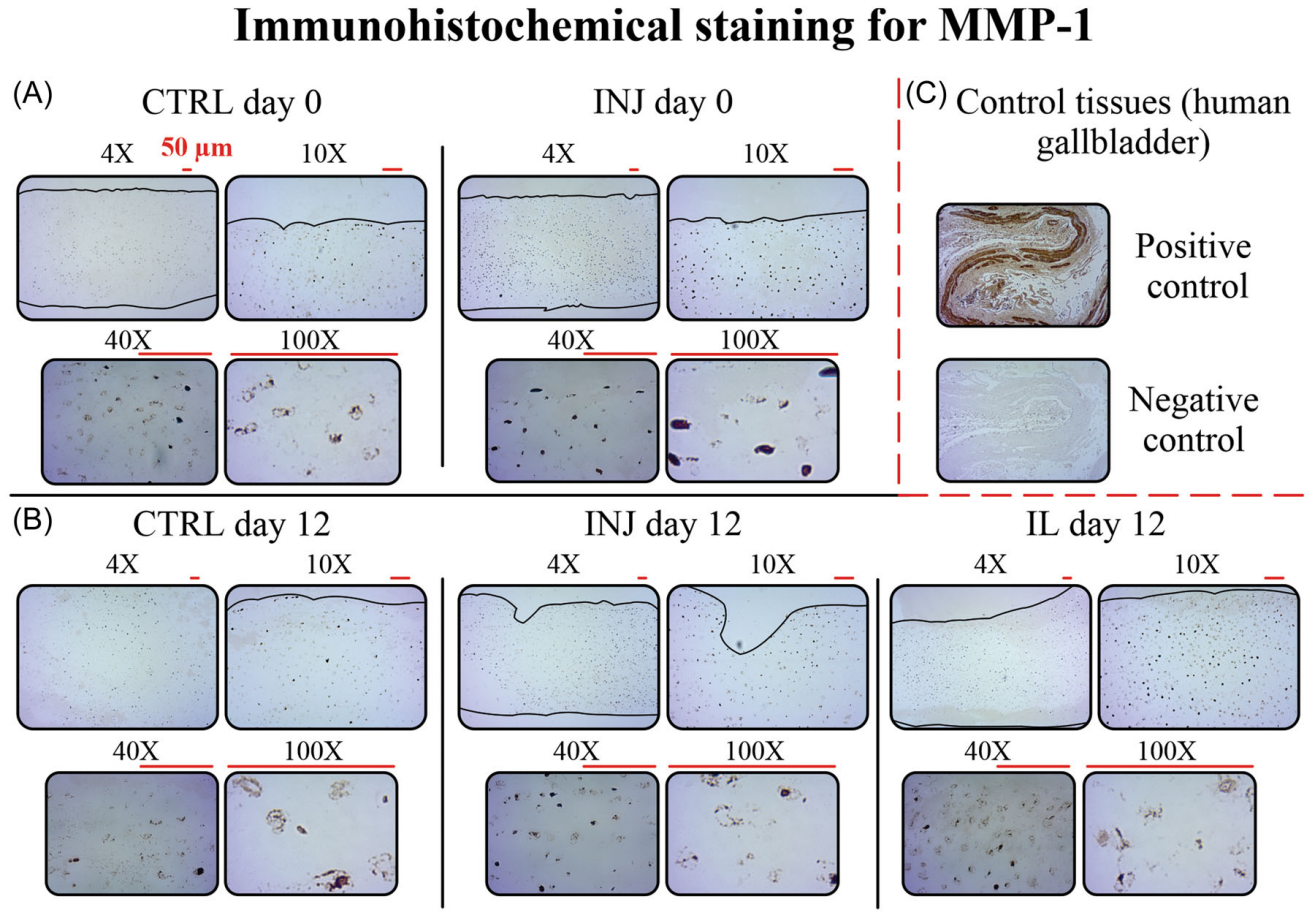
Qualitative images of immunohistochemical staining for MMP‐1 from articular cartilage sections on day 0 and day 12 for CTRL, INJ, and IL groups. Each group is shown with representative samples of four different magnifications (4×, 10×, 40×, and 100×). The 10×, 40×, and 100× images were captured from the most superficial part of each section. (A) More intense MMP‐1 staining was observed in INJ group on day 0 compared to CTRL along tissue depth. (B) Similarly, more intense staining was observed in INJ and IL groups compared to CTRL on day 12. The staining intensity in INJ and IL groups on day 12 was lower compared to INJ group on day 0. (C) For the validation of the staining protocol, positive and negative control sections of human gallbladder tissue were used. MMP, matrix metalloproteinase; CTRL, free‐swelling control; INJ, injurious loading; IL, interleukin‐1α.

**Figure 9:**
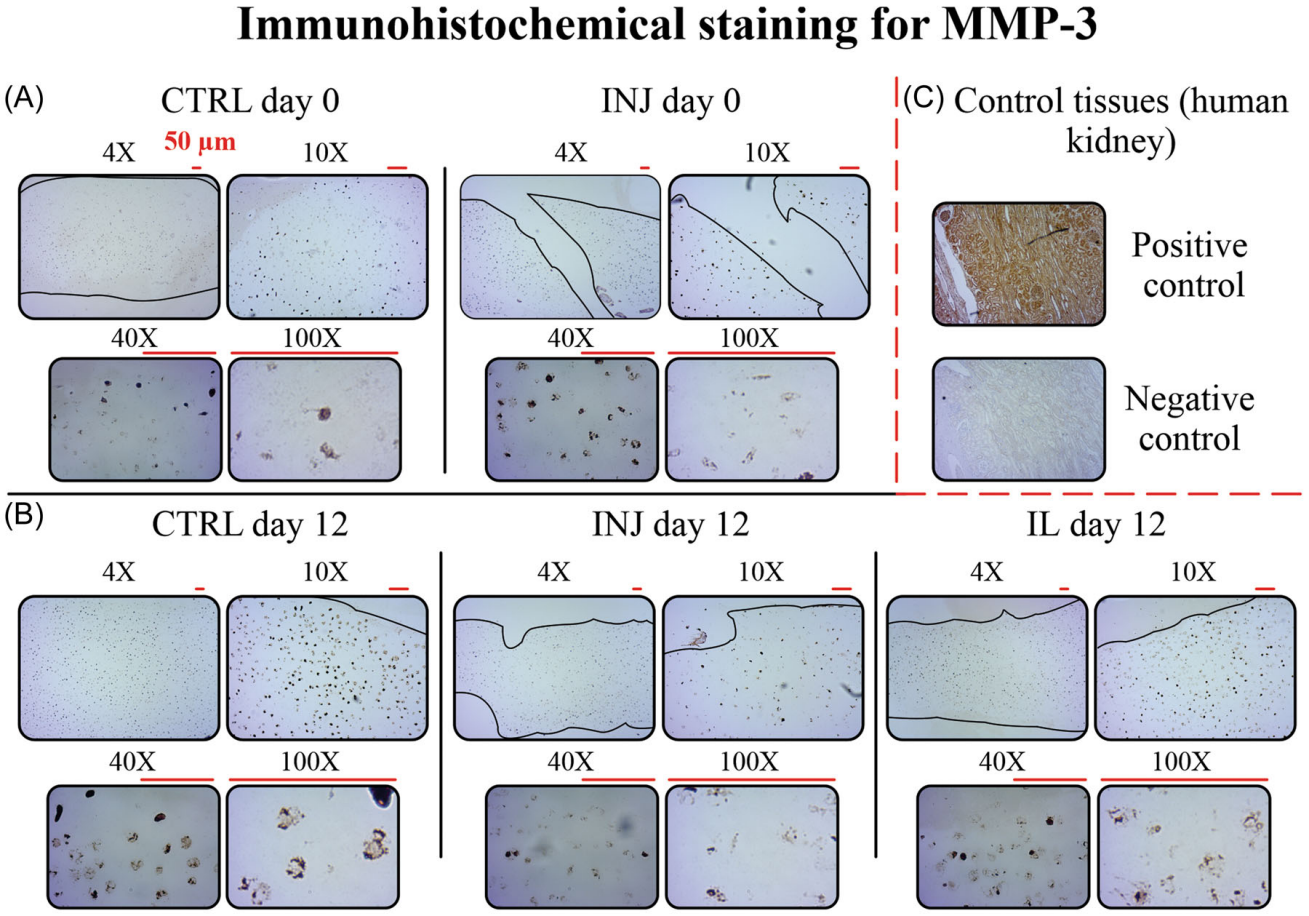
Qualitative images of immunohistochemical staining for MMP‐3 from articular cartilage sections on day 0 and day 12 for CTRL, INJ, and IL groups. Each group is shown with representative samples of four different magnifications (4×, 10×, 40×, and 100×). The 10×, 40×, and 100× images were captured from the most superficial part of each section. (A) More intense MMP‐3 staining was observed in INJ group on day 0 compared to CTRL at the deeper regions of tissue. (B) On day 12, INJ group represented less staining intensity compared to CTRL. The staining intensity was lower in INJ and IL groups on day 12 compared to INJ group on day 0. (C) For the validation of the staining protocol, positive and negative control sections of human kidney tissue were used. MMP, matrix metalloproteinase; CTRL, free‐swelling control; INJ, injurious loading; IL, interleukin‐1α.

We observed positive immunoreactivity of the MMP proteins in the studied tissues, with qualitatively more intense staining for MMP‐1 and MMP‐3 localized within cell lacunae throughout the tissue depth in the INJ group compared to CTRL group on day 0 (Figures 8A and 9A). On day 12, IL group exhibited the highest MMP‐1 staining intensity along depth compared to INJ and CTRL groups (Figure 8B). On the other hand, INJ and IL groups showed lower MMP‐3 staining intensities compared to CTRL group on day 12 (Figure 9B). For both proteases, staining intensities in INJ and IL groups were lower on day 12 compared to INJ group on day 0.

## 4 DISCUSSION

We established an in vitro explant culture model of early‐stage PTOA to investigate spatially the effect of injurious loading, IL‐1α‐challenge, and moderate cyclic loading on collagen content and integrity in young bovine cartilage explants on day 0 and day 12 post injury. The results supported our hypothesis that injurious loading can result in lower collagen content near cartilage lesions when compared to away from lesions. To our surprise, this already occurred at the baseline (day 0). Such loss of collagen content near lesions was amplified by day 12. In contrast to our hypothesis, the presence of inflammation or cyclic loading did not potentiate the loss of collagen content. However, moderate cyclic loading showed potential positive effects (less collagen loss) in the intact areas. On the other hand, we did not observe any changes in collagen integrity within or between any groups, reflecting the possibility of no collagen denaturation near lesion in response to injurious loading, IL‐1α‐challenge, and cyclic loading. In addition to possible direct mechanical rupture of fibrils, our immunohistochemical analyses suggest that release of MMP‐1,3 enzymes could partially explain the time‐dependent, depth‐wise decrease in collagen content.

Previous findings of Thibault et al. suggested that an excessive level of loading (~35% strain amplitude, 22%/s strain rate, cyclically loaded for 17 min) can cause rapid (~within minutes) denaturation of collagen fibrils (~loss of collagen integrity), followed later by subsequent release of collagen fragments (~loss of collagen content) into the culture medium (~within days).^6^ Also, Chen et al. demonstrated an increase in the percentage of denatured collagens in canine cartilage explants over 2 days in culture, following cyclical impact loading (5 MPa, for 120 min).^7^ The highest level of denaturation was found near the formed surface lesions. While our results align with this previous literature regarding the localized loss of collagen content, we did not observe any differences in collagen integrity (~denaturation) between or within groups. The variation between studies’ conclusions regarding collagen denaturation may stem from the differences in the in vitro experimental designs. First, Thibault et al. and Chen et al. utilized mature cartilage samples, while immature cartilage explants were employed in our study. Mature cartilage is characterized by higher mechanical stiffness and nonuniform collagen fibril network structure.^40^, ^41^, ^42^ Such differences in the mechanical and structural properties could play a substantial role on the collagen fibrils’ response to mechanical loading (i.e., the extent of denaturation or collagen loss could differ between immature and mature cartilage under same loading conditions). Secondly, both earlier studies used different mechanical overloading protocols compared to the injurious loading protocol used in our study. For example, Thibault et al. mentioned that their mechanical protocol represents excessive level of loading, yet still physiological (i.e., not traumatic).^6^ On the other hand, our mechanical loading was designed to be harmful and cause lesions on the cartilage surface (one cycle with 50% strain amplitude). Therefore, our results could be specific to the loading protocol used.

The early loss of collagen content near lesion reported in our work could support the role of mechanical overloading directly causing localized rupture and failure of collagen fibrils and subsequent outburst of collagen fragments from the tissue in the vicinity of lesion.^6^, ^7^, ^19^, ^43^ On the other hand, our previous study revealed the presence of early localized cell death near lesions on day 3, reaching deeper tissue regions by day 12 in injuriously loaded cartilage.^13^, ^20^, ^31^ Additionally, there are several pieces of evidence from previous literature suggesting the presence of cell damage in response to tissue overloading.^6^, ^7^, ^19^, ^43^ Cell death and cell damage could in turn later lead to a substantial increase in the production and release of MMPs, leading to a prolonged enzymatic degradation of the collagen fibrils.^44^, ^45^ Thus, the intensified collagen loss near lesions and the extension of collagen loss along tissue depth on day 12 reported in our work could be partly caused by the MMP‐driven enzymatic degradation.^44^

To qualitatively test our hypothesis of MMP‐driven enzymatic cleavage of collagen fibrils, we applied IHC staining for MMP‐1 and MMP‐3 on cartilage sections. Both proteases have been shown to cause cleavage of collagen I, II, III, and IX.^38^, ^39^, ^45^, ^46^ The results support our hypothesis with increased MMP‐1 and MMP‐3 staining intensity localized near cells in response to injurious loading (day 0). This intense protease staining on the day of injury may explain the later depth‐dependent loss of collagen content in INJ group compared to CTRL group on day 12 (Figure 6). Additionally, the elevated MMP‐1 staining in IL group on day 12 could corroborate the role of IL‐1 in activating latent MMPs.^16^, ^47^ This might then highlight that injurious loading and IL‐1 treatment can both lead to the upregulation of MMPs and the enzymatic cleavage of collagen fibrils but potentially through different pathways.^30^, ^45^, ^48^, ^49^, ^50^

Previous studies, with similar in vitro setups as in our work, reported reduced aggrecan content near cartilage lesions as early as 3 days post injury.^13^, ^15^ A gradual decrease in aggrecan content was also found at intact regions starting from day 3.^13^, ^31^ We hypothesize that the early localized fracture of collagen fibrils, leading to lesion formation, may have contributed to localized aggrecan release through the lesion edges.^13^ On the other hand, aggrecan is believed to play a role in the protection of collagen fibrils from enzymatic degradation,^16^, ^18^ as it was suggested that aggrecan molecules could be binding on the surface of collagens, coating and protecting the fibrils from proteases.^18^ The early aggrecan loss reported near lesion and at intact regions (from day 3) could be playing a role in aggravating subsequent (day 12) MMP‐driven loss of collagen along tissue depth.

Next, we aimed to examine the combined effect of mechanical injury and IL‐1α‐challenge to confirm our hypothesis that simultaneous application of both treatments would potentiate the decrease in collagen content beyond the effects of each treatment alone.^16^, ^45^, ^51^ Our hypothesis was motivated by previous literature reporting that synergistic effect of both injurious loading and cytokines such as TNF‐α, IL‐6, and IL‐1 can cause increased proteoglycan loss, cell apoptosis, and decreased cell biosynthesis compared to injury or cytokine treatment applied alone.^13^, ^17^, ^20^ Our results refuted our hypothesis, showing no differences in collagen content when comparing INJ + IL to INJ and IL groups. The inability of INJ + IL to potentiate the decrease in collagen content suggests that injury or cytokines alone could elicit the tissue to a near‐maximum catabolic response for collagen fibrils. This trend of maximum catabolic cell response was also observed in a recent human cartilage proteomic study where explants were subjected to injurious loading (60% strain amplitude, 300%/s strain rate), and IL‐6 and TNF‐α treatments.^28^ Their results revealed that injury or cytokines alone could substantially decrease the synthesis of collagen II, and the combination of both did not cause further decrease within 21 days.^28^ Inconsistencies in the results between different studies can be expected due to different loading regimens, and doses and types of inflammatory cytokines.^12^, ^14^, ^15^

Notably, our findings are from controlled in vitro studies, where PTOA is experimentally induced with injurious loading and high cytokine levels to observe disease‐like changes within feasible timeframe. However, in physiological in vivo conditions, effects of injurious loading, cytokines, and their combination on collagen loss and damage are most likely subject‐specific and manifested at a broader timeframe than 12 days.^9^

Our depth‐dependent collagen content comparisons suggest that the application of moderate cyclic loading can potentially slow down the decrease of collagen content caused by mechanical injury and/or IL‐1α treatment (Figure 7). However, our results also indicate that the used cyclic loading protocol did not have any effect on collagen content near lesions (Figure 4A). In previous studies, cyclic loading could tune down cytokine‐driven fibrillar matrix degradation, reduce cell death, and increase cell biosynthesis.^13^, ^20^, ^31^ Additionally, cyclic loading was previously able to retain the aggrecan content locally at the deeper regions of the cartilage explants subjected to IL‐1α treatment and injurious loading.^13^ Our results could highlight the protective capability of cyclic loading against collagen enzymatic degradation by the mechanical tensile straining of the fibrils.^52^, ^53^ Alternatively, in regions where collagen fibrils were damaged by mechanical rupture due to injurious loading, such as near lesions (loss of collagen content on day 0, Figure 4), the protective effect of cyclic loading might be negligible.^52^ The role of cyclic loading and the fibril strain levels needed to modulate cartilage degradation could be confirmed in the future with the help of computational modeling of injured cartilage.^54^

Some animal models and in vitro studies suggest that aggrecan loss is the first sign of cartilage degradation and precedes collagen fibril damage and collagen content loss in early PTOA^15^, ^18^, ^55^, ^56^ One suggestion was that the reduction in proteoglycan content contributes to the decrease of tissue stiffness which may lead to further tissue damage and later cause localized decrease in collagen content.^55^ On the other hand, our results demonstrate rapid loss of collagen content (day 0) after cartilage injury, yet such loss was localized near lesions. This indicates that the localized loss of collagen content near lesions could be another early sign of the onset of PTOA.

The current study focused on assessing changes in total collagen content without discerning between the collagen types as we aimed to investigate the general collagen fibril network response to injury. However, earlier studies have demonstrated that FTIR could differentiate between collagen types I and II.^57^ We cannot rule out the possibility of increase of collagen type I and decrease of type II collagen after injury (as observed in fibrocartilage).^58^ To gain a more comprehensive understanding of collagen type‐specific compositional changes in cartilage, future research should combine immunohistochemical assessments or enzyme‐linked immunosorbent assays with the FTIR analysis.^59^, ^60^ Additionally, investigating tissue‐level and local collagen organization, fibril diameter, d‐period, and interfibrillar spacing with electron and atomic force microscopy^61^, ^62^ and with polarized light microscopy^63^ could reveal potential alterations in the collagen ultrastructure, complementing the compositional changes observed here.

We utilized cartilage explants from Experiment #2 to include two more groups in our study (CL and INJ + CL) as we aimed to expand our investigations of the potential beneficial effect of moderate cyclic loading in mitigating collagen loss in injured cartilage. We acknowledge that the slight change in timing of the cyclic loading and resting periods might have a minor effect on cartilage degradation. However, literature evidence suggests that loading strain and loading rate would have major effect on cartilage health, while resting period timing should not affect the results.^13^, ^20^, ^31^ Such negligible effect was supported with our results indicating no difference between CL groups along tissue depth regardless of the used protocol.

To conclude, our study suggests that the localized collagen content loss near lesions is initiated rapidly after the mechanical rupture of cartilage and this loss is further intensified after 12 days. On the other hand, moderate cyclic loading is suggested to slow down the collagen content loss in the intact regions of the tissue. Such information offers new insights on the early spatial‐dependent effect of traumatic injury that could lead to cartilage degradation. Additionally, our work demonstrates a possible protective role by physiological cyclic loading after the injury. The experimental data we provide in this work will be essential to validate computational models of cartilage degeneration after injury.^31^, ^64^, ^65^ Such models may reveal detailed mechanobiological mechanisms leading to collagen loss post injury and provide a tool to predict PTOA progression and effectiveness of therapies.

## AUTHOR CONTRIBUTIONS

Moustafa Hamada: Study design, data analysis, data acquisition, interpretation, manuscript writing and revision, Atte S. A. Eskelinen: Study design, data analysis, data acquisition, interpretation, manuscript writing, and revision, Cristina Florea: Data acquisition, interpretation, manuscript writing, and revision, Santtu Mikkonen: Data Analysis, manuscript revision, Petteri Nieminen: Data acquisition, interpretation, manuscript revision, Alan J. Grodzinsky: Study design, interpretation, manuscript writing, and revision, Petri Tanska: Study design, interpretation, manuscript writing, and revision, Rami K. Korhonen: Study design, interpretation, manuscript writing and revision. All authors approved the final submitted manuscript.
